# Curbing Inflammation in Multiple Sclerosis and Endometriosis: Should Mast Cells Be Targeted?

**DOI:** 10.1155/2015/452095

**Published:** 2015-10-15

**Authors:** David A. Hart

**Affiliations:** ^1^Department of Surgery, Wound Healing Initiative, University of Calgary, Calgary, AB, Canada; ^2^Centre for Hip Health & Mobility, University of British Columbia, Vancouver, BC, Canada

## Abstract

Inflammatory diseases and conditions can arise due to responses to a variety of external and internal stimuli. They can occur acutely in response to some stimuli and then become chronic leading to tissue damage and loss of function. While a number of cell types can be involved, mast cells are often present and can be involved in the acute and chronic processes. Recent studies in porcine and rabbit models have supported the concept of a central role for mast cells in a “nerve-mast cell-myofibroblast axis” in some inflammatory processes leading to fibrogenic outcomes. The current review is focused on the potential of extending aspects of this paradigm into treatments for multiple sclerosis and endometriosis, diseases not usually thought of as having common features, but both are reported to have activation of mast cells involved in their respective disease processes. Based on the discussion, it is proposed that targeting mast cells in these diseases, particularly the early phases, may be a fruitful avenue to control the recurring inflammatory exacerbations of the conditions.

## 1. Introduction

Induction of acute inflammation can result from a wide variety of stimuli ranging from infection by microorganisms to tissue injury. Such inflammation can be beneficial and lead to elimination of the microorganisms, as well as healing of injured tissues. In most circumstances, the induction of inflammation involves cells of the innate or non-antigen-specific immune system, and the inflammation subsides when the insult is eliminated either passively or actively via molecules such as resolvins (reviewed in [1]) and other mechanisms. The extent of the inflammation is dependent in part on the extent of the tissue damage (endogenous cells such as tissue macrophages, mast cells versus recruitment of circulating cells such as PMN, monocytes, mast cells, and lymphocytes) and the nature of the insult.

Certainly, mast cells are known to be involved in a variety of normal and disease processes including acute inflammation (reviewed in [2]), allergic responses (reviewed in [3, 4]), and chronic autoimmunity (reviewed in [5, 6]). While much focus has been on the role of mast cells in acute reactions, it is clear that these cells also play important roles in diverse chronic conditions.

Acute inflammatory responses can become chronic in some instances, possibly due to host susceptibility (e.g., genetics) or other factors, leading to a prolonged response which does not resolve on its own. Thus, abnormalities at the induction or resolution stages could lead to a chronic inflammatory state with associated fibrosis, pain, and loss of tissue function depending on which tissue is affected (e.g., kidney, brain, reproductive organs, lung, and joint tissues). In some susceptible hosts, the chronic inflammatory state is associated with the induction of autoimmunity (autoantibodies and self-reactive T-lymphocytes) which can then participate in further target tissue damage and loss of function. In preclinical models, it is possible to induce autoimmunity in a susceptible host (usually mice and rats) which can then mimic aspects of the disease, but such studies may not be relevant to the initial disease process or even the exacerbations of tissue damage (discussed in more detail below). Even in “natural” forms of some conditions (e.g., the NZB/W model of SLE), one can protect target organ integrity without overtly blocking the autoantibody profile ([7–9]; reviewed in [10]) and, therefore, separate potential inflammatory stimuli (e.g., autoantibodies) from actual target tissue damage (e.g., the kidney). Therefore, inducing immune-specific autoimmunity may not accurately reflect the mechanisms of initial disease induction and its progression in models of chronic inflammatory diseases such as murine RA, MS, and other conditions.

Recent studies have focused on abnormal inflammatory responses associated with skin wound healing in a pig model [11–13] and inflammatory responses associated with joint injuries leading to joint contractures in both humans [14, 15] and rabbits [16, 17]. In both the pig and rabbit models, a response to tissue injury leads to a hypertrophic-like healing response in the red Duroc pig model [11–13] and joint contractures in the rabbit model [16, 17]. In the pig model, a unique “biphasic” inflammatory process was detected after skin injury, while in the rabbit model it appeared that the initial injuries to the joint capsule and bone led to a chronic inflammatory phenotype accompanied by a fibrotic thickening of the capsule. Interestingly, the profile of postinjury changes in the rabbit was very similar to those detected in tissue from humans with elbow contractures [14, 15]. Of note was the consistent pattern of increased nerve elements, mast cells, and myofibroblasts in the abnormal phenotype of healing (reviewed in [18, 19]). These abnormal healing patterns have led to the concept of “a nerve-mast cell-myofibroblast axis” in such conditions (reviewed in [18, 19]), with neuroinflammation a potential key element of dysregulated responses to injury (reviewed in [18–20]). It is clear that nerves are important in normal healing (reviewed in [21, 22]), and that abnormal neural activation could result in fibrotic outcomes.

In view of the above abnormal patterns of responses to tissue injury, the use of the mast cell stabilizer ketotifen to interfere with healing after injury in both the pig skin injury model [13] and the rabbit model of joint contractures [16, 17] was studied. In these models, ketotifen treatment was able to convert the abnormal healing in the pig model to a more normal phenotype and in the rabbit joint contracture model an inhibition of joint contraction formation. In the porcine model, ketotifen treatment had to be initiated immediately after injury as delayed initiation of treatment did not impact the abnormal phenotype [13]. Interestingly, ketotifen treatment of animals in both models led to declines in the numbers of nerves, mast cells, and myofibroblasts in the injured tissues, potentially indicating that the postulated “nerve-mast cell-myofibroblast axis” is not unidirectional and it is interactive in nature.

Ketotifen has been used in the treatment of asthma for >20 years and its safety and efficacy are well known (discussed in [16, 17]). Thus, a pilot clinical trial of ketotifen use in preventing joint contractures in patients with elbow injuries has been initiated, as well as assessment of serum biomarkers associated with risk of contractures initiated (in progress).

The above findings raised the possibility that ketotifen (or other mast cell stabilizers) may have efficacy in other diseases with a known neuroinflammatory component and mast cell involvement. Ketotifen has been used in scleroderma, but the results were not positive in spite of some initial results (discussed in [23]). However, it may have been used too late in the skin disease processes, but it may have efficacy in those patients that are at risk of developing pulmonary complications of scleroderma (discussed in [23]).

Two other such conditions where ketotifen may have efficacy, based on what is known about mast cell involvement, are multiple sclerosis and endometriosis. In both conditions, mast cells have been implicated as being present and/or activated during active disease.

## 2. Multiple Sclerosis

### 2.1. Etiology of Multiple Sclerosis (MS)

The etiology of MS is currently unknown, but a viral etiology has been debated and assessed for decades (reviewed in [24, 25]). This has been based in part by the known increased incidence in temperate zones of the northern hemisphere. Interestingly, some evidence for increased incidence based on time of the year when a person is born has been reported [26], possibly implicating vitamin D levels as well (discussed in [27–30]). Furthermore, females have a higher incidence of the disease than males (reviewed in [31]), and the occurrence of relapses is diminished during pregnancy (reviewed in [31]). Interestingly, this finding is not unlike what is also known to occur in rheumatoid arthritis patients where ~70% of pregnant females with RA experience a remission of the disease during pregnancy. Also similar to RA patients, many female MS patients experience a reactivation of the disease during the postpartum period [31]. As inflammatory processes are dampened during pregnancy, this pattern implicates some common mechanisms being involved.

Several of the viruses that have been implicated are many that are known, and the disease then associated with being a susceptible individual for autoimmunity. Others, such as retroviruses activated by unknown stimuli (possibly environmental), have recently been postulated to be involved in disease initiation (discussed in [32, 33]). Once disease has been initiated, it is not clear whether the continued presence of a virus is critical.

MS, like other autoimmune diseases such as rheumatoid arthritis, can be varied in disease progression, with a remitting-relapsing progressive phenotype being the most common. The different subtypes of MS may involve different immune and other elements and have different biomarker signatures (discussed in [34]). Patients with MS are also at risk of loss of mobility and falls; the latter are also a risk factor for fractures which could further reduce mobility and long term independent living [35, 36].

### 2.2. Treatment of MS

A number of treatment options have been explored for patients with MS. These include interferons, specific monoclonal antibodies, and other immune-modulating drugs which focus on the autoimmune aspects of the condition primarily. Recently, the potential vascular aspects of the condition were addressed following much publicity [37, 38], but again the clinical trials did not yield outcomes in support of the concept.

Additional studies have advanced the concept that matrix metalloproteinases (MMPs) should be targeted in MS [39–42], but such proteinases serve a variety of normal functions throughout the body, and the use of specific MMP inhibitors in other conditions such as osteoarthritis has been discontinued after considerable investment by industry. Thus, MMP inhibition may not be a fruitful direction to follow in MS for long term control of the disease, but such approaches could provide some insights into the downstream influences related to tissue damage mechanisms.

The potential use of stem cells to modulate degenerative processes and repair damaged tissues in MS and other neurological diseases has not yet met with much success. The initial discoveries of stem cells in the brain were met with much fanfare and promise, but again after much investment by government agencies and industry, >20 years later that promise has not yet been fulfilled in spite of considerable effort (reviewed in [43–46]). One reason for this may be that such stem cells cannot function well to repair damaged tissues in MS in the face of a chronic inflammatory disease [47]. Therefore, it may be important to control the disease activity effectively and then use stem cells from any number of tissue sources to facilitate repair (e.g., similar to what is needed in rheumatoid arthritis [48] or osteoarthritis [49, 50]). As stem cells from a number of tissue sources or induced pluripotent stem cells can differentiate into neural-like cells, it remains unclear whether brain-derived stem cells offer any unique capacities in this regard. Interestingly, some recent reports indicate that human embryonic neural stem cells may have efficacy in some unique murine models of MS [51].

Finally, attempts are being made to initiate repair of tissue damage in MS (reviewed in [43, 52]). Such studies are still in an early state, but without controlling the inflammatory processes prior to initiating repair modalities, this may not yield the results hoped for. Interestingly, a clinical trial of the antihistamine clemastine fumarate (over-the-counter Tavist) in relapsing-remitting MS has been initiated in an attempt to promote remyelination, but the study is still ongoing (ClinicalTrials.gov). One of the limitations of trying to initiate repair of established MS lesions is the fibrotic nature of the “scar-like” material deposited in such lesions as a consequence of the inflammation (reviewed in [53]). In spite of limitations in removing established fibrotic deposits, some authors have advocated targeting the plasminogen activator system to remove or inhibit the process [54].

Another mitigating variable that may influence the ability to modulate inflammation relates to the potential epigenetic changes that could occur as the MS becomes more chronic, similar to what has been reported in other chronic inflammatory diseases such as RA (discussed in [55]). Thus, early disease may respond to approaches to curb inflammation that are not as effective later in the disease course.

### 2.3. Mast Cells in MS

Mast cells are found in the brain in association with a number of cells and on the brain side of the blood-brain barrier (reviewed in [56]). Activation of mast cells is believed to play an important role in neuroinflammation, including diseases such as MS, as well as events such as brain trauma and stroke (reviewed in [57]). Furthermore, stress which is known to influence MS disease activity has been reported to involve mast cells (reviewed in [58]). In rat models, acute stress also leads to disruption of the blood-brain barrier via activation of mast cells [59]. Interestingly, in the report by Esposito et al. [59], the response to stress could be mitigated by administration of the mast cell stabilizer, disodium cromoglycate.

Elevated levels of mast cell products have been detected in fluids from MS patients. These include mast cell tryptase in CSF from MS patients [60] and histamine levels in CSF from MS patients [61, 62]. In spite of such evidence, the role of mast cells in MS is still somewhat controversial [63], in part because some mouse models of MS, such as induced EAE, have indicated that while mast cells can accumulate in EAE, they are “dispensable” [64]. Of course the limitation here is that it is an induced model of “MS” and in mice, which may not reflect the human condition very well. Being induced via active immunization with neural antigens is very artificial and likely is bypassing the natural initiating steps and events associated with the relapsing-remitting or primary-progressive phenotypes. Furthermore, the outcomes may be influenced by the genetics of the mouse strain used in such studies [65], thus adding a further complication to defining of the role of mast cells in mouse models. In spite of the controversies around their role, some authors have advocated for the targeting of mast cells in autoimmune diseases such as MS [66, 67].

### 2.4. Targeting Mast Cells in MS

From the above discussion, it is fairly clear that there is a potential role for targeting mast cells in MS, either alone or in combination with other interventions, in order to control disease activity. As mentioned above, some preclinical models have indicated use of a mast cell stabilizer (disodium cromoglycate) alleviated some aspects of the EAE disease in rodents [59]. Our own work in chronic fibrosis models [13, 16, 17] showed that a different mast cell stabilizer, ketotifen, was effective in abrogating abnormal mast cell activity but did not affect normal healing in porcine and rabbit models. Ketotifen has been used extensively in the treatment of asthma (reviewed in [18, 19]), effective doses in both paediatric and adult populations are known, as well as the safety profile, and the oral administration of the drug should also enhance compliance. Interestingly, the drug effectively crosses the blood-brain barrier [68], and therefore that obstacle should not be an issue.

While no evidence for specific mast cell targeted agents in the treatment of MS was detected in the literature, it was noted that a recent drug that was approved for treatment of the remitting-relapsing form of the disease, dimethyl fumarate (discussed in [69]), was shown to induce mast cell apoptosis in vitro [70]. Of the multiple variants of the drug, only the dimethyl fumarate form was effective in inducing apoptosis of human HMC-1 cells and cord blood derived-mast cells. Therefore, the effectiveness of this drug in inhibiting mast cell involvement in MS in vivo should likely be further investigated. Of interest is the fact that the drug used in previous studies from the author's laboratory was ketotifen fumarate [13, 16, 17], but whether this was relevant to effectiveness is currently unknown. In addition, the antihistamine clemastine fumarate is being used in an ongoing clinical trial in relapsing-remitting MS (ClinicalTrials.gov).

The real question related to targeting mast cells in MS is when to administer the drug (early disease or later), and whether to use such interventions to curb inflammation should be provided as part of a cocktail to target more than one aspect of the disease. As these are complex diseases, it is likely that curbing inflammation via targeting mast cells would be a component of a “cocktail” aimed at controlling disease activity in a proactive manner. Without more complete control of disease activity via a multipronged approach, it may be somewhat optimistic to expect repair of the damage with new interventions in MS or other autoimmune diseases (reviewed in [48]). Finally, it is unclear why such approaches have not already advanced to the clinical trial stage. Either they have occurred and were not reported due to failure (the system moves slowly most of the time) or there is no commercial incentive as many of these drugs are off patent protection and are being “repurposed.”

Finally, it should be clear regarding outcome expectations during attempts to curb inflammation by targeting mast cells. Firstly, such treatment targeting mast cells may preferentially impact development of new MS lesions rather than resolution of established lesions. However, “reactivation” of established lesions may be inhibited from progressing. Based on previous work in porcine fibrogenic models, the drug has to be administered early after injury and was ineffective if provided after fibrosis is established ([13]; reviewed in [23]). As the time course for “remitting-relapsing” forms of MS is somewhat unpredictable with regard to timing, this may mean that trials focused on curbing mast cell activation should be sufficiently long to accurately allow assessment of efficacy. Secondly, one cannot assume that the nerve-mast cell “axis” is unidirectional. Thus, use of mast cell stabilizers in fibrogenic situations led to decreased numbers of both mast cells and nerve elements [13, 18, 19, 23]. The caveat here is that the fibrogenic situation during wound healing involves peripheral nerve elements and not central brain neurons and related cells. It is not known if this would influence the outcomes. Finally, it should be clearly stated that curbing inflammation by targeting mast cells is downstream from the inciting events which likely come from the neural elements themselves and may be due to virus infections or retroviral activation or some other mechanisms (discussed earlier). Therefore, targeting mast cells in this disease may address some of the mechanisms related to lesions/damage, but not the actual basis for the primary dysfunction. However, if effective in this regard, it may allow for better understanding of some of the root causes. If positive results are obtained, then perhaps other neurodegenerative diseases, such as Alzheimer's disease, may also be targeted.

## 3. Endometriosis (EMS)

**Figure 1:**
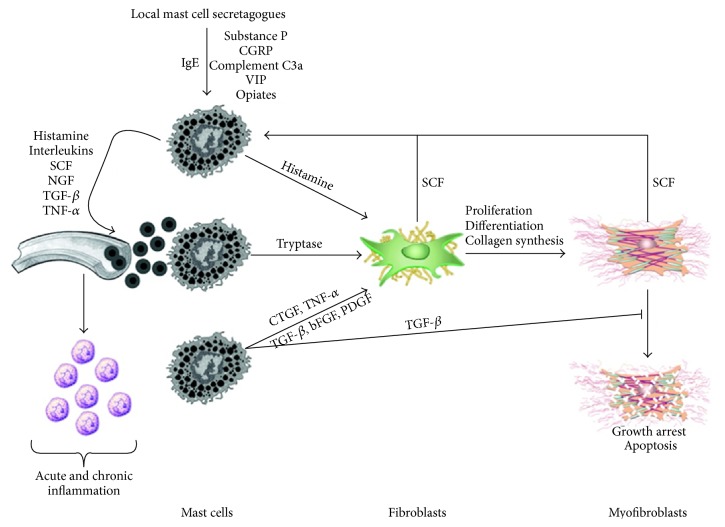
Mast cells mediated inflammation and fibrosis. Mast cells circulate as CD34-positive precursor cells and terminally differentiate in connective tissues. Both IgE dependent and independent mechanisms can activate mast cells causing the release of preformed and newly synthesized proinflammatory mediators. Many of these mediators increase vascular permeability and promote the recruitment of other inflammatory cells and additional mast cell precursors. SCF is also secreted by activated fibroblasts and myofibroblasts, further potentiating mast cell recruitment and proliferation. TGF-*β* is a potent fibroblast mitogen and stimulator of myofibroblast differentiation. It also impedes myofibroblasts apoptosis. bFGF, basic fibroblast growth factor; CGRP, calcitonin gene-related peptide; CTGF, connective tissue growth factor; NGF, nerve growth factor; PDGF, platelet-derived growth factor; SCF, stem cell factor; TGF-*β*, transforming growth factor-beta; TNF-*α*, tumor necrosis factor-alpha; VIP, vasoactive intestinal peptide. This figure was reproduced from [19].

A second disease/condition that also appears to involve neuroinflammation with mast cell involvement is endometriosis (EMS). EMS is clearly an estrogen-dependent chronic inflammatory disorder (reviewed in [71–73]). Thus, females with fluctuating estrogen levels and this condition experience chronic effects for many years. While usually not thought of in conjunction with MS, there are some parallels that exist that indicate some common elements (e.g., neuroinflammation, mast cells, fibrosis, and loss of function) are present in both conditions and, as such, raise the possibility that some of these common elements could be therapeutic targets (e.g., mast cells) (Figure 1). This figure (Figure 1) describing how such common features lead to fibrotic outcomes can also be found in Monument et al. [19] and in the web version of that article at http://www.liebertpub.com/wound. EMS, MS, and the fibrogenic skin wound healing and joint contractures discussed above may use some unique features, as well as the common elements leading to fibrosis, but mast cells are likely central to all of these conditions.

### 3.1. Etiology of EMS

Endometriosis is defined as the extrauterine growth of endometrial tissue which results in pain and infertility in ~10–15% of females (reviewed in [71–76]). One theory regarding how the condition arises relates to viable endometrial cells entering the peritoneum via retrograde menstruation, implantation, and establishment of ectopic growths (discussed in [76]). Such growths become extensively innervated and vascularized and contain mast cells (reviewed in [71–73]). However, other theories have also been raised regarding the origins of the tissues, and the controversy is still not resolved (discussed in [71, 77]). A recent report has also implicated endometrial mesenchymal stem/progenitor cells in development of the disease [78]. Some reports also indicate that there may be some genetic risk in the development of EMS (discussed in [79]). Also similar to MS, vitamin D may or may not be involved in EMS [80].

Similarly, but in a somewhat difference context, fibrosis is a feature of endometriosis [81, 82], as well as proteinases such as MMPs ([83]; reviewed in [84]), and others [85]. Genetic polymorphisms in some MMP genes such as MMP-1, as well as others, have been implicated in endometriosis [86]. Interestingly, variants of the MMP-1 promoter can be influenced by estrogen [87].

The pain aspects of the condition appear to be related to fluctuating hormonal influences during the menstrual cycle, with associated swelling and inflammation (reviewed in [88]). EMS is considered a hormone-dependent inflammatory disease/condition. Several reports indicate that the inflammatory response is altered in EMS [89], potentially due to its chronicity which can lead to epigenetic alterations (reviewed in [90]). However, in some preclinical models, EMS can continue in ovariectomized animals, thus implicating other mechanisms (e.g., the innate immune system) in disease progression [91]. Which inflammatory pathways are involved in EMS and how they are regulated by sex hormones have been the subject of considerable research (reviewed in [92, 93]).

There are some reports indicating that EMS is an autoimmune disease or at least has some features of an autoimmune disease ([94]; reviewed in [95]). Whether the autoimmune aspects are a cause or a consequence of the chronic inflammation in a susceptible individual remains unclear. What is clear is that it is a chronic inflammatory condition that does not resolve. Interestingly, peritoneal fluid from patients with EMS contains higher levels of some cytokines than those without this condition (e.g., MCP-1 and IL-8 [96]). Similar to MS, stress can exacerbate symptoms of EMS in preclinical models [97].

### 3.2. Treatment of EMS

EMS can be treated conservatively using NSAIDS to control the pain [98], use of oral contraceptives to manipulate the hormonal environment (discussed in [99]), or surgery to remove the lesions (reviewed in [100, 101]). Surgical removal of lesions is effective in the short term, but lesions may reappear [101]. Other authors have suggested more innovative treatments such as gene therapy [102] or targeting angiogenesis which is prominent in EMS [103]. Recently, the use of exercise has been promoted to impact EMS symptoms, but this has not been validated (reviewed in [104]). In preclinical models, vitamin D and a plant-derived anti-inflammatory molecule, beta-caryophyllene inhibited growth of EMS lesions and induced regressions of implants [105, 106].

As surgery in endometriosis to remove lesions is only partially successful and lesions reappear, some effort to inhibit reformation of lesion by inhibiting adhesion formation has been attempted. However, such trials have not been overtly successful thus far [107].

### 3.3. Mast Cells in EMS

Mast cells play a role in a number of normal processes in females. These include involvement in reproduction, pregnancy, and labour (reviewed in [108]). Therefore, one has to be clear regarding what is an abnormal involvement in a process versus variation in a normal process.

Mast cells are prominent in EMS tissue but whether their role(s) in EMS development and progression are central to the disease or peripheral with mast cells being drawn to the site of lesions is still being debated (discussed in [109]). However, some evidence would indicate that the mast cells are more central to lesion development and progression [110]. Degranulated mast cells have been detected in EMS lesions [111] and activated mast cells implicated in the associated fibrosis [112]. Furthermore, mast cells have been reported to express estrogen receptors and thus should be responsive to the sex hormones [113]. In fact, estrogen has been reported to result in mast cell activation with release of mediators, in part due to interactions with ER-alpha [113]. Furthermore, mast cells in EMS lesions are commonly found in close approximation to neural elements in such lesions (discussed in [109, 114]). Such close approximation of these two elements in lesions is somewhat supportive of the concept of active neuroinflammation and pain in EMS [115] and is analogous to what we have observed in abnormal skin wound healing and joint contraction models previously that were responsive to mast cell stabilizer interventions [13, 16, 17].

### 3.4. Targeting Mast Cells in EMS

Several targets to influence EMS progression have focused on aromatase inhibitors, anti-TNF-alpha, and dienogest (discussed in [100] and others) to address the known cytokine and hormonal influences on the condition. In addition, given the above discussed evidence for the active involvement of mast cells in EMS, it is not surprising that some authors have also suggested targeting such cells to alleviate pain and progression of EMS lesions. D'Cruz and Uckun [116] have suggested using Janus kinase 3 inhibitors to interfere with EMS pain and lesion progression. Janus kinase 3 is well expressed by mast cells and is, therefore, a likely potential target. It is not clear from the literature whether this suggestion has progressed to preclinical models or the clinic. Interestingly, women with endometriosis who were treated with a levonorgestrel-releasing intrauterine system had fewer mast cells in their endometrial biopsies but not in normal peritoneum [117]. However, the mechanisms involved have not been identified (e.g., direct effects versus indirect consequences of treatment). Interestingly, a somewhat recent report by Zhu and Zhang [115] has postulated that nerves release peptides such as Substance P that can induce mast cell degranulation with subsequent release of bioactive molecules that could contribute to the pain of endometriosis. Such a scenario in EMS is consistent with recent in vitro studies indicating that Substance P can enhance mast cell stimulation of myofibroblast activity [118].

While mast cells have been suggested as a potential target in EMS, from the literature searched, no evidence for the use of mast cell stabilizers such as ketotifen or disodium cromoglycate in this condition could be detected. Given the extensive safety and use profiles of such drugs in asthma and allergic conditions, this is somewhat surprising. Furthermore, one might have expected some serendipity with regard to these drugs due to taking the drugs for asthma and also having EMS in a younger female population.

Whatever the basis for not having this suggestion being pursued is, given the recent success in other circumstances (e.g., abnormal skin wound healing and joint contracture prevention), more thought should be given to implement some pilot studies to assess the efficacy of these drugs in EMS.

As with MS, if indeed mast cell directed drugs/approaches are attempted in EMS, then one will have to carefully match expectations and outcomes. Also as with MS, mast cells in EMS may be downstream from an inciting event (e.g., neurostimulation/excitation), or they could be more central via estrogen/hormone activation of the mast cells as a more primary event. Therefore, the relationship between treatment and appropriate outcome measures will need to be carefully monitored. Secondly, EMS, like MS, is a complex set of conditions, and therefore mast cell directed agents may need to be matched with complementary approaches to yield a multipronged approach to effectively control different aspects of the condition as there may be multiple control points and some redundancies in the disease processes.

## 4. Summary and Conclusions

MS and EMS share a number of parallels in the disease process (e.g., neuroinflammation, fibrosis, proteinase activation, and mast cells). They are not necessarily unique in this regard and other conditions (e.g., hypertrophic scarring after burns, pulmonary fibrosis) may also share some of these features (reviewed/discussed in [23]). However, MS and EMS are two diseases in which mast cells are believed to be intimately involved, but no targeting of these cells has been attempted based on a review of the available literature. As potential drugs targeting mast cells, such as ketotifen and disodium cromoglycate, have well documented safety and efficacy profiles, they could be used initially for pilot studies and then larger multicenter studies without need for a number of safety trials. Interestingly, both of the drugs named are off patent protection, a fact that would potentially make studies less costly but also be less of an incentive for industry support. However, implementing some pilot studies to curb inflammation with mast cell directed agents is likely warranted.

While such trials with ketotifen and related drugs are likely warranted, it should be pointed out that chronic long term suppression of mast cell function could have health consequences in some individuals and such drugs are likely not without some potential side effects in subsets of patients (e.g., adverse events or events associated with chronic impairment of normal mast cell functions). Thus, any trials initiated should carefully monitor potential side effect issues. However, some patients with neurofibroma have been treated with ketotifen for 30 years with continued beneficial effects and no reported overt side effects [119].

It should also be reiterated that the “nerve-mast cell-myofibroblast axis” (reviewed in [19, Figure 1]) is supported by in vitro [120] and in vivo [13, 16, 17] studies, but the in vivo studies indicate that such an axis is likely not unidirectional as treatment with ketotifen led to declines in all components of the postulated axis that would not be consistent with a unidirectional axis leading to fibrosis. Therefore, either there are as yet undefined influences of the drug on nerves and/or myofibroblasts or the axis functions in vivo in a bidirectional fashion. Thus, evidence exists for how such an axis could function, but evidence for why it is activated remains to be determined and individual disease entities may have different “whys” such processes are initiated and exacerbated.

Finally, as discussed above, it is clear that EMS is an estrogen-dependent disease; while MS may be more prevalent in females, it has not been shown to be estrogen-dependent. Conversely, MS occurs in a naturally neural-rich environment, while EMS does not, but the abnormal tissue does have nerves. Thus, the “triggers” for activation of MS versus EMS may be different, but they do share common elements including mast cell involvement. Furthermore, both MS [121] and EMS [120] are not uniform diseases but are heterogeneous conditions with patient subsets with differing characteristics or disease courses. Thus, some patients may respond to mast cell stabilizers more effectively than others, and such considerations should be kept in mind when designing future studies.

## References

[1] Cash J. L., Norling L. V., Perretti M. (2014). Resolution of inflammation: targeting GPCRs that interact with lipids and peptides. *Drug Discovery Today*.

[2] Theoharides T. C., Alysandratos K.-D., Angelidou A. (2012). Mast cells and inflammation. *Biochimica et Biophysica Acta: Molecular Basis of Disease*.

[3] Harvima I. T., Levi-Schaffer F., Draber P. (2014). Molecular targets on mast cells and basophils for novel therapies. *Journal of Allergy and Clinical Immunology*.

[4] da Silva E. Z. M., Jamur M. C., Oliver C. (2014). Mast cell function: a new vision of an old cell. *Journal of Histochemistry and Cytochemistry*.

[5] Christy A. L., Brown M. A. (2007). The multitasking mast cell: positive and negative roles in the progression of autoimmunity. *Journal of Immunology*.

[6] Walker M. E., Hatfield J. K., Brown M. A. (2012). New insights into the role of mast cells in autoimmunity: evidence for a common mechanism of action?. *Biochimica et Biophysica Acta—Molecular Basis of Disease*.

[7] Krause G., Zhao P., Martin L., Fritzler M., Benediktsson H., Hart D. A. (1992). LiCl prolongs survival and alters disease progression in the NZB/W model of systemic lupus erythematosus. *Lithium*.

[8] Lenz S. P., Izui S., Hart D. A. (1997). Evidence that lithium chloride treatment of female NZB/W mice does not influence autoantibody profiles in this murine model of systemic lupus erythematosus. *Journal of Trace and Microprobe Techniques*.

[9] Hart D. A., Kydd A., Sciore P., Reno C. (2000). Lithium treatment of NZB/W F1 and parental mice leads to alterations in kidney mRNA levels: further evidence for genetic and gender influences on Li responsiveness. *Journal of Trace and Microprobe Techniques*.

[10] Hart D. A., Fritzler M. J., Lucas K. C., Becker R. W., Lucas K. C., Gallicchio V. S. (1999). Lithium chloride mediated enhancement of survival in murine models. Characterization of autoimmune disease: evidence that Li influences target organ resistence to immune insults. *The Biological Actions of Lithiumn: New Perspective*.

[11] Gallant-Behm C. L., Olson M. E., Hart D. A. (2006). Cytokine and growth factor mRNA expression patterns associated with the hypercontracted, hyperpigmented healing phenotype of red duroc pigs: a model of abnormal human scar development?. *Journal of Cutaneous Medicine and Surgery*.

[12] Gallant-Behm C. L., Reno C., Tsao H., Hart D. A. (2006). Characterization of genetic involvement in skin wound healing in domestic pigs: assessment of molecular expression patterns in [Yorkshire x red Duroc] x Yorkshire backcross animals. *Journal of Investigative Dermatology*.

[13] Gallant-Behm C. L., Hildebrand K. A., Hart D. A. (2008). The mast cell stabilizer ketotifen prevents development of excessive skin wound contraction and fibrosis in red Duroc pigs. *Wound Repair and Regeneration*.

[14] Hildebrand K. A., Zhang M., Hart D. A. (2005). Evidence for a high rate of matrix turnover in chronic human elbow contractures. *Clinical Orthopaedics and Related Research*.

[15] Hildebrand K. A., Zhang M., Hart D. A. (2007). Myofibroblast up-regulators are elevated in joint capsules in post-traumatic contractures. *Clinical Orthopaedics and Related Research*.

[16] Monument M. J., Hart D. A., Befus A. D., Salo P. T., Zhang M., Hildebrand K. A. (2010). The mast cell stabilizer ketotifen fumarate lessens contracture severity and myofibroblast hyperplasia: a study of a rabbit model of posttraumatic joint contractures. *The Journal of Bone & Joint Surgery—American Volume*.

[17] Monument M. J., Hart D. A., Befus A. D., Salo P. T., Zhang M., Hildebrand K. A. (2012). The mast cell stabilizer ketotifen reduces joint capsule fibrosis in a rabbit model of post-traumatic joint contractures. *Inflammation Research*.

[18] Monument M. J., Hart D. A., Salo P. T., Befus A. D., Hildebrand K. A. (2013). Posttraumatic elbow contractures: targeting neuroinflammatory fibrogenic mechanisms. *Journal of Orthopaedic Science*.

[19] Monument M. J., Hart D. A., Salo P. T., Befus A. D., Hildebrand K. A. (2015). Neuroinflammatory mechanisms of connective tissue fibrosis: targeting neurogenic and mast cell contributions. *Advances in Wound Care*.

[20] Hart D. A., Frank C. B., Bray R. C., Gordon S. L., Blair S. J., Fine L. J. (1995). Inflammatory processes in repetitive motion and over-use syndromes: potential role of neurogenic inflammation mechanisms in tendons and ligaments. *Repetitive Motion Disorders of the Upper Extremity*.

[21] Ackermann P. W., Salo P. T., Hart D. A. (2009). Neuronal pathways in tendon healing. *Frontiers in Bioscience*.

[22] Ackermann P. W., Hart D. A. (2013). Influence of comorbidities: neuropathy, vasculopathy, and diabetes on healing response quality. *Advances in Wound Care*.

[23] Hart D. A. (2013). Treatments for fibrosis development and progression: lessons learned from preclinical models and potential impact on human conditions such as scleroderma, pulmonary fibrosis, hypertrophic scarring and tendinopathies. *Journal of Biomedical Science and Engineering*.

[24] Owens G. P., Gilden D., Burgoon M. P., Yu X., Bennett J. L. (2011). Viruses and multiple sclerosis. *Neuroscientist*.

[25] Virtanen J. O., Jacobson S. (2012). Viruses and multiple sclerosis. *CNS & Neurological Disorders—Drug Targets*.

[26] Dobson R., Giovannoni G., Ramagopalan S. (2013). The month of birth effect in multiple sclerosis: systematic review, meta-analysis and effect of latitude. *Journal of Neurology, Neurosurgery and Psychiatry*.

[27] Hewer S., Lucas R., van der Mei I., Taylor B. V. (2013). Vitamin D and multiple sclerosis. *Journal of Clinical Neuroscience*.

[28] James E., Dobson R., Kuhle J., Baker D., Giovannoni G., Ramagopalan S. V. (2013). The effect of vitamin D-related interventions on multiple sclerosis relapses: a meta-analysis. *Multiple Sclerosis*.

[29] Salzer J., Biström M., Sundström P. (2014). Vitamin D and multiple sclerosis: where do we go from here?. *Expert Review of Neurotherapeutics*.

[30] Tizaoui K., Berraies A., Hamdi B., Kaabachi W., Hamzaoui K., Hamzaoui A. (2015). Association between vitamin D receptor polymorphisms and multiple sclerosis: systematic review and meta-analysis of case-control studies. *Cellular & Molecular Immunology*.

[31] Miller D. H., Fazekas F., Montalban X., Reingold S. C., Trojano M. (2014). Pregnancy, sex and hormonal factors in multiple sclerosis. *Multiple Sclerosis*.

[32] Hon G. M., Erasmus R. T., Matsha T. (2013). Multiple sclerosis-associated retrovirus and related human endogenous retrovirus-W in patients with multiple sclerosis: a literature review. *Journal of Neuroimmunology*.

[33] Nissen K. K., Laska M. J., Hansen B. (2013). Endogenous retroviruses and multiple sclerosis-new pieces to the puzzle. *BMC Neurology*.

[34] Amin B., Maurer A., Voelter W., Melms A., Kalbacher H. (2014). New potential serum biomarkers in multiple sclerosis identified by proteomic strategies. *Current Medicinal Chemistry*.

[35] Finlayson M. L., Peterson E. W., Asano M. (2014). A cross-sectional study examining multiple mobility device use and fall status among middle-aged and older adults with multiple sclerosis. *Disability and Rehabilitation: Assistive Technology*.

[36] Hoang P. D., Cameron M. H., Gandevia S. C., Lord S. R. (2014). Neuropsychological, balance, and mobility risk factors for falls in people with multiple sclerosis: a prospective cohort study. *Archives of Physical Medicine and Rehabilitation*.

[37] Pullman D., Zarzeczny A., Picard A. (2013). Media, politics and science policy: MS and evidence from the CCSVI trenches. *BMC Medical Ethics*.

[38] Hankey G. J., Sandercock P., Cantisani T. A., Celani M. G. (2014). Is the 'liberation procedure' for multiple sclerosis really liberating?. *Journal of Neurology, Neurosurgery and Psychiatry*.

[39] Yong V. W. (1999). The potential use of MMP inhibitors to treat CNS diseases. *Expert Opinion on Investigational Drugs*.

[40] Yong V. W., Agrawal S. M., Stirling D. P. (2007). Targeting MMPs in acute and chronic neurological conditions. *Neurotherapeutics*.

[41] Agrawal S. M., Lau L., Yong V. W. (2008). MMPs in the central nervous system: where the good guys go bad. *Seminars in Cell and Developmental Biology*.

[42] Mirshafiey A., Asghari B., Ghalamfarsa G., Jadidi-Niaragh F., Azizi G. (2014). The significance of matrix metalloproteinases in the immunopathogenesis and treatment of multiple sclerosis. *Sultan Qaboos University Medical Journal*.

[43] Ben-Hur T. (2011). Cell therapy for multiple sclerosis. *Neurotherapeutics*.

[44] Huang J. K., Franklin R. J. M. (2012). Current status of myelin replacement therapies in multiple sclerosis. *Progress in Brain Research*.

[45] Grade S., Bernardino L., Malva J. O. (2013). Oligodendrogenesis from neural stem cells: perspectives for remyelinating strategies. *International Journal of Developmental Neuroscience*.

[46] Tullis G. E., Spears K., Kirk M. D. (2014). Immunological barriers to stem cell therapy in the central nervous system. *Stem Cells International*.

[47] Ben-Hur T., Ben-Menachem O., Furer V., Einstein O., Mizrachi-Kol R., Grigoriadis N. (2003). Effects of proinflammatory cytokines on the growth, fate, and motility of multipotential neural precursor cells. *Molecular and Cellular Neuroscience*.

[48] Hart D. A., Kydd A. S., Frank C. B., Hildebrand K. A. (2004). Tissue repair in rheumatoid arthritis: challenges and opportunities in the face of a systemic inflammatory disease. *Best Practice & Research: Clinical Rheumatology*.

[49] Ando W., Heard B. J., Chung M., Nakamura N., Frank C. B., Hart D. A. (2012). Ovine synovial membrane-derived mesenchymal progenitor cells retain the phenotype of the original tissue that was exposed to in-vivo inflammation: evidence for a suppressed chondrogenic differentiation potential of the cells. *Inflammation Research*.

[50] Harris Q., Seto J., O'Brien K. (2013). Monocyte chemotactic protein-1 inhibits chondrogenesis of synovial mesenchymal progenitor cells: an in vitro study. *Stem Cells*.

[51] Chen L., Coleman R., Leang R. (2014). Human neural precursor cells promote neurologic recovery in a viral model of multiple sclerosis. *Stem Cell Reports*.

[52] Ben-Hur T., Fainstein N., Nishri Y. (2013). Cell-based reparative therapies for multiple sclerosis. *Current Neurology and Neuroscience Reports*.

[53] Fernández-Klett F., Priller J. (2014). The fibrotic scar in neurological disorders. *Brain Pathology*.

[54] Gur-Wahnon D., Mizrachi T., Maaravi-Pinto F.-Y. (2013). The plasminogen activator system: involvement in central nervous system inflammation and a potential site for therapeutic intervention. *Journal of Neuroinflammation*.

[55] Bottini N., Firestein G. S. (2013). Epigenetics in rheumatoid arthritis: a primer for rheumatologists. *Current Rheumatology Reports*.

[56] Silver R., Curley J. P. (2013). Mast cells on the mind: new insights and opportunities. *Trends in Neurosciences*.

[57] Nelissen S., Lemmens E., Geurts N. (2013). The role of mast cells in neuroinflammation. *Acta Neuropathologica*.

[58] Karagkouni A., Alevizos M., Theoharides T. C. (2013). Effect of stress on brain inflammation and multiple sclerosis. *Autoimmunity Reviews*.

[59] Esposito P., Gheorghe D., Kandere K. (2001). Acute stress increases permeability of the blood-brain-barrier through activation of brain mast cells. *Brain Research*.

[60] Rozniecki J. J., Hauser S. L., Stein M., Lincoln R., Theoharides T. C. (1995). Elevated mast cell tryptase in cerebrospinal fluid of multiple sclerosis patients. *Annals of Neurology*.

[61] Tuomisto L., Kilpeläinen H., Riekkinen P. (1983). Histamine and histamine-N-methyltransferase in the CSF of patients with multiple sclerosis. *Agents and Actions*.

[62] Kallweit U., Aritake K., Bassetti C. L. (2013). Elevated CSF histamine levels in multiple sclerosis patients. *Fluids and Barriers of the CNS*.

[63] Costanza M., Colombo M. P., Pedotti R. (2012). Mast cells in the pathogenesis of multiple sclerosis and experimental autoimmune encephalomyelitis. *International Journal of Molecular Sciences*.

[64] Bennett J. L., Blanchet M.-R., Zhao L. (2009). Bone marrow-derived mast cells accumulate in the central nervous system during inflammation but are dispensable for experimental autoimmune encephalomyelitis pathogenesis. *The Journal of Immunology*.

[65] Sayed B. A., Walker M. E., Brown M. A. (2011). Cutting edge: mast cells regulate disease severity in a relapsing-remitting model of multiple sclerosis. *Journal of Immunology*.

[66] Zappulla J. P., Arock M., Mars L. T., Liblau R. S. (2002). Mast cells: new targets for multiple sclerosis therapy?. *Journal of Neuroimmunology*.

[67] Brown M. A., Hatfield J. K. (2012). Mast cells are important modifiers of autoimmune disease: with so much evidence, why is there still controversy?. *Frontiers in Immunology*.

[68] Tashiro M., Mochizuki H., Sakurada Y. (2006). Brain histamine H_1_ receptor occupancy of orally administered antihistamines measured by positron emission tomography with ^11^C-doxepin in a placebo-controlled crossover study design in healthy subjects: a comparison of olopatadine and ketotifen. *British Journal of Clinical Pharmacology*.

[69] Nicholas J. A., Boster A. L., Imitola J., O'Connell C., Racke M. K. (2014). Design of oral agents for the management of multiple sclerosis: benefit and risk assessment for dimethyl fumarate. *Drug Design, Development and Therapy*.

[70] Förster A., Preussner L. M., Seeger J. M. (2013). Dimethylfumarate induces apoptosis in human mast cells. *Experimental Dermatology*.

[71] Burney R. O., Giudice L. C. (2012). Pathogenesis and pathophysiology of endometriosis. *Fertility and Sterility*.

[72] Vercellini P., Viganò P., Somigliana E., Fedele L. (2014). Endometriosis: pathogenesis and treatment. *Nature Reviews Endocrinology*.

[73] Ahn S. H., Monsanto S. P., Miller C., Singh S. S., Thomas R., Tayade C. (2015). Pathophysiology and immune dysfunction in endometriosis. *BioMed Research International*.

[74] Hickey M., Ballard K., Farquhar C. (2014). Endometriosis. *British Medical Journal*.

[75] Kobayashi H., Higashiura Y., Shigetomi H., Kajihara H. (2014). Pathogenesis of endometriosis: the role of initial infection and subsequent sterile inflammation (Review). *Molecular Medicine Reports*.

[76] Silva A. B., Srivastava P., Shivaji S. (2014). Understanding the pathogenesis of endometriosis through proteomics: recent advances and future prospects. *Proteomics and Clinical Applications*.

[77] Vinatier D., Orazi G., Cosson M., Dufour P. (2001). Theories of endometriosis. *European Journal of Obstetrics & Gynecology and Reproductive Biology*.

[78] Pittatore G., Moggio A., Benedetto C., Bussolati B., Revelli A. (2014). Endometrial adult/progenitor stem cells: pathogenetic theory and new antiangiogenic approach for endometriosis therapy. *Reproductive Sciences*.

[79] Burney R. O. (2013). The genetics and biochemistry of endometriosis. *Current Opinion in Obstetrics and Gynecology*.

[80] Sayegh L., Fuleihan G. E.-H., Nassar A. H. (2014). Vitamin D in endometriosis: a causative or confounding factor?. *Metabolism: Clinical and Experimental*.

[81] Chegini N. (2008). TGF-*β* system: the principal profibrotic mediator of peritoneal adhesion formation. *Seminars in Reproductive Medicine*.

[82] Matsuzaki S., Darcha C. (2013). Involvement of the Wnt/beta-catenin signaling pathway in the cellular and molecular mechanisms of fibrosis in endometriosis. *PLoS ONE*.

[83] Pitsos M., Kanakas N. (2009). The role of matrix metalloproteinases in the pathogenesis of endometriosis. *Reproductive Sciences*.

[84] Young V. J., Brown J. K., Saunders P. T. K., Horne A. W. (2013). The role of the peritoneum in the pathogenesis of endometriosis. *Human Reproduction Update*.

[85] Osuga Y., Hirota Y., Yoshino O., Hirata T., Koga K., Taketani Y. (2012). Proteinase-activated receptors in the endometrium and endometriosis. *Frontiers in Bioscience*.

[86] de Marqui A. B. T. (2012). Genetic polymorphisms and endometriosis: contribution of genes that regulate vascular function and tissue remodeling. *Revista da Associação Médica Brasileira*.

[87] Achari Y., Lu T., Hart D. A. (2008). Polymorphisms in the promoter regions for human MMP-1 and MMP-13 lead to differential responses to the alpha and beta isoforms of estrogen receptor and their ligand in vitro. *Biochimica et Biophysica Acta: Molecular Basis of Disease*.

[88] Bruner-Tran K. L., Herington J. L., Duleba A. J., Taylor H. S., Osteen K. G. (2013). Medical management of endometriosis: emerging evidence linking inflammation to disease pathophysiology. *Minerva Ginecologica*.

[89] Khoufache K., Michaud N., Harir N., Kibangou Bondza P., Akoum A. (2012). Anomalies in the inflammatory response in endometriosis and possible consequences: a review. *Minerva Endocrinologica*.

[90] Izawa M., Taniguchi F., Terakawa N., Harada T. (2013). Epigenetic aberration of gene expression in endometriosis. *Frontiers in Bioscience*.

[91] Khan K. N., Kitajima M., Fujishita A., Nakashima M., Masuzaki H. (2013). Toll-like receptor system and endometriosis. *Journal of Obstetrics and Gynaecology Research*.

[92] King A. E., Critchley H. O. D. (2010). Oestrogen and progesterone regulation of inflammatory processes in the human endometrium. *Journal of Steroid Biochemistry and Molecular Biology*.

[93] Maybin J. A., Critchley H. O. D., Jabbour H. N. (2011). Inflammatory pathways in endometrial disorders. *Molecular and Cellular Endocrinology*.

[94] Gajbhiye R., Suryawanshi A., Khan S. (2008). Multiple endometrial antigens are targeted in autoimmune endometriosis. *Reproductive BioMedicine Online*.

[95] Eisenberg V. H., Zolti M., Soriano D. (2012). Is there an association between autoimmunity and endometriosis?. *Autoimmunity Reviews*.

[96] Oral E., Olive D. L., Arici A. (1996). The peritoneal environment in endometriosis. *Human Reproduction Update*.

[97] Cuevas M., Flores I., Thompson K. J., Ramos-Ortolaza D. L., Torres-Reveron A., Appleyard C. B. (2012). Stress exacerbates endometriosis manifestations and inflammatory parameters in an animal model. *Reproductive Sciences*.

[98] Zito G., Luppi S., Giolo E. (2014). Medical treatments for endometriosis-associated pelvic pain. *BioMed Research International*.

[99] Hee L., Kettner L. O., Vejtorp M. (2013). Continuous use of oral contraceptives: an overview of effects and side-effects. *Acta Obstetricia et Gynecologica Scandinavica*.

[100] Kim S. H., Chae H. D., Kim C.-H., Kang B. M. (2013). Update on the treatment of endometriosis. *Clinical and Experimental Reproductive Medicine*.

[101] Yeung P. (2014). The laparoscopic management of endometriosis in patients with pelvic pain. *Obstetrics and Gynecology Clinics of North America*.

[102] Shubina A. N., Egorova A. A., Baranov V. S., Kiselev A. V. (2013). Recent advances in gene therapy of endometriosis. *Recent Patents on DNA & Gene Sequences*.

[103] Djokovic D., Calhaz-Jorge C. (2014). Angiogenesis as a therapeutic target in endometriosis. *Acta Medica Portuguesa*.

[104] Bonocher C. M., Montenegro M. L., Rosa e Silva J. C., Ferriani R. A., Meola J. (2014). Endometriosis and physical exercises: a systematic review. *Reproductive Biology and Endocrinology*.

[105] Abbas M. A., Taha M. O., Zihlif M. A., Disi A. M. (2013). *β*-Caryophyllene causes regression of endometrial implants in a rat model of endometriosis without affecting fertility. *European Journal of Pharmacology*.

[106] Abbas M. A., Taha M. O., Disi A. M., Shomaf M. (2013). Regression of endometrial implants treated with vitamin D_3_ in a rat model of endometriosis. *European Journal of Pharmacology*.

[107] Somigliana E., Vigano P., Benaglia L., Busnelli A., Vercellini P., Fedele L. (2012). Adhesion prevention in endometriosis: a neglected critical challenge. *The Journal of Minimally Invasive Gynecology*.

[108] Menzies F. M., Shepherd M. C., Nibbs R. J., Nelson S. M. (2011). The role of mast cells and their mediators in reproduction, pregnancy and labour. *Human Reproduction Update*.

[109] Kirchhoff D., Kaulfuss S., Fuhrmann U., Maurer M., Zollner T. M. (2012). Mast cells in endometriosis: guilty or innocent bystanders?. *Expert Opinion on Therapeutic Targets*.

[110] Paula R., Oliani A. H., Vaz-Oliani D. C. M., D’Ávila S. C. G. P., Oliani S. M., Gil C. D. (2014). The intricate role of mast cell proteases and the annexin A1-FPR1 system in abdominal wall endometriosis. *Journal of Molecular Histology*.

[111] Sugamata M., Ihara T., Uchiide I. (2005). Increase of activated mast cells in human endometriosis. *American Journal of Reproductive Immunology*.

[112] Konno R., Yamada-Okabe H., Fujiwara H. (2003). Role of immunoreactions and mast cells in pathogenesis of human endometriosis—morphologic study and gene expression analysis. *Human Cell*.

[113] Zaitsu M., Narita S.-I., Lambert K. C. (2007). Estradiol activates mast cells via a non-genomic estrogen receptor-*α* and calcium influx. *Molecular Immunology*.

[114] Anaf V., Chapron C., El Nakadi I., De Moor V., Simonart T., Noël J.-C. (2006). Pain, mast cells, and nerves in peritoneal, ovarian, and deep infiltrating endometriosis. *Fertility and Sterility*.

[115] Zhu L., Zhang X. (2013). Research advances on the role of mast cells in pelvic pain of endometriosis. *Zhejiang Da Xue Xue Bao Yi Xue Ban*.

[116] D'cruz O. J., Uckun F. M. (2007). Targeting mast cells in endometriosis with Janus kinase 3 inhibitor, JANEX-1. *American Journal of Reproductive Immunology*.

[117] Engemise S. L., Willets J. M., Emembolu J. O., Konje J. C. (2011). The effect of the levonorgestrel-releasing intrauterine system, Mirena on mast cell numbers in women with endometriosis undergoing symptomatic treatment. *European Journal of Obstetrics Gynecology and Reproductive Biology*.

[118] Hildebrand K. A., Zhang M., Befus A. D., Salo P. T., Hart D. A. (2014). A myofibroblast-mast cell-neuropeptide axis of fibrosis in post-traumatic joint contractures: an in vitro analysis of mechanistic components. *Journal of Orthopaedic Research*.

[119] Riccardi V. M. (2015). Ketotifen suppression of NF1 neurofibroma growth over 30 years. *American Journal of Medical Genetics Part A*.

[120] Wang G., Tokushige N., Markham R., Fraser I. S. (2009). Rich innervation of deep infiltrating endometriosis. *Human Reproduction*.

[121] McKay K. A., Kwan V., Duggan T., Tremlett H. (2015). Risk factors associated with the onset of relapsing-remitting and primary progressive multiple sclerosis: a systematic review. *BioMed Research International*.

